# Effect of the Internet Commerce on Dispersal Modes of Invasive Alien Species

**DOI:** 10.1371/journal.pone.0099786

**Published:** 2014-06-16

**Authors:** Magdalena Lenda, Piotr Skórka, Johannes M. H. Knops, Dawid Moroń, William J. Sutherland, Karolina Kuszewska, Michał Woyciechowski

**Affiliations:** 1 Institute of Environmental Sciences, Jagiellonian University, Krakow, Poland; 2 Institute of Zoology, Poznan University of Life Sciences, Poznan, Poland; 3 School of Biological Sciences, University of Nebraska, Lincoln, Nebraska, United States of America; 4 Institute of Systematics and Evolution of Animals, Polish Academy of Sciences, Kraków, Poland; 5 Conservation Science Group, Department of Zoology, University of Cambridge, Cambridge, United Kingdom; University of Tartu, Estonia

## Abstract

The spread of invasive alien plants has considerable environmental and economic consequences, and is one of the most challenging ecological problems. The spread of invasive alien plant species depends largely on long-distance dispersal, which is typically linked with human activity. The increasing domination of the internet will have impacts upon almost all components of our lives, including potential consequences for the spread of invasive species. To determine whether the rise of Internet commerce has any consequences for the spread of invasive alien plant species, we studied the sale of thirteen of some of the most harmful Europe invasive alien plant species sold as decorative plants from twenty-eight large, well known gardening shops in Poland that sold both via the Internet and through traditional customer sales. We also analyzed temporal changes in the number of invasive plants sold in the largest Polish internet auction portal. When sold through the Internet invasive alien plant species were transported considerably longer distances than for traditional sales. For internet sales, seeds of invasive alien plant species were transported further than were live plants saplings; this was not the case for traditional sales. Also, with e-commerce the shape of distance distribution were flattened with low skewness comparing with traditional sale where the distributions were peaked and right-skewed. Thus, e-commerce created novel modes of long-distance dispersal, while traditional sale resembled more natural dispersal modes. Moreover, analysis of sale in the biggest Polish internet auction portal showed that the number of alien specimens sold via the internet has increased markedly over recent years. Therefore internet commerce is likely to increase the rate at which ecological communities become homogenized and increase spread of invasive species by increasing the rate of long distance dispersal.

## Introduction

Species invasions are both a result and cause of global ecological changes. Numerous studies have shown that invasive alien plant species can often establish and change edaphic conditions in new habitats, change their structure or even create novel invasive-dominated ecosystems [1]–[8]. However, a key factor is invasive species dispersal; species can only become successful invaders if they can disperse to suitable habitats.

Most plant dispersal is over a short range and it has a right skewed leptokurtic distribution, with a tail of few individuals travelling very long distances [9], [10]. These few long distance dispersers are important in colonization of new areas and in determining the rate of geographical range spread [10], [11]. This tail is the most important factor shaping the invasion mechanisms of alien plant species. The long tail of extreme movements by invasive species is often attributable to various forms of human transportation, which allows alien species to cross geographical barriers and to colonize new localities as well as escape from lag phase of colonization [5], [12]–[15].

Just as some alien species benefitted from the development of the train network in the UK, such as Oxford Ragwort, whose seeds were carried along the tracks after the trains [16], alien species currently benefit from the worldwide development of vehicle, navy and aerial transportation [12]–[15]. In recent years much of the trade has been through internet sales, with an associated transportation network [17], [18]. However, we barely understand how the relatively new global phenomenon of internet commerce may affect the dispersal modes of invasive alien plants at landscape or regional scales.

Currently, many alien plant species, including invasive ones, are available for sale in many garden shops [19], [20]. The traditional commercial model in shops or floral markets comprises sales to visiting customers, which limits the type of transportation, and distances on which alien plant species are transported. However, the recent rise in internet sales have included the development of trade in animals and plants to customers who may not even know where the shop is located [21], [22]. Thus, the internet trade is much less constrained in transportation type and distance by which alien invasive species may travel from shop to customer. Consequently, the distribution of distances that are travelled by alien species from a shop to a customer should be less peaked and much less skewed (be more Gaussian-like distribution) than in case of traditional sale, assuming that buyers in the internet are located at random distances from the shop. There are currently few studies that show how internet commerce enhances purchase of invasive species, and so increases propagule pressure of invasive species [22]. There is thus a need to determine how internet commerce affects dispersal modes and the functioning of populations in the environment on landscape or regional scales and how this compares with the traditional shop trade.

The aim of this study was to characterize the role of the internet sale in the long distance dispersal and spread of invasive species. The study was carried out in Poland, where the internet and traditional commerce of garden flowering plants are both relatively well developed. The following hypotheses were tested:

1. Internet trade results in larger movement distances of invasive plant species than does traditional trading, where much of the trade will be to local people.
2. Internet trade is less constrained by the distance and generates distribution of dispersal distances that are less peaked and less skewed than in more limited traditional trading.
3. Seeds are transported on longer distances than seedlings. Seedlings are known to be more fragile and more likely to be collected in person than sent by post.
4. Supply and demand of invasive alien species sold via internet auctions is growing over time indicating increased interest in alien invasive species, what suggests also higher propagule pressure of exotic species.

## Materials and Methods

We chose 13 invasive species that are widespread in Poland, some of which are amongst the most harmful invasive plant species in Europe [23], and which were easily available through garden shops and internet auctions. The following species were studied: *Acer negundo*, *Buddleia davidii*, *Echinocystis lobata*, *Elodea canadensis*, *Impatiens glandulifera*, *Prunus serotina*, *Quercus rubra*, *Rhus typhina*, *Robinia pseudoacacia*, *Rosa rugosa*. We also examined the following genera that are rarely distinguishing at the species level so can be sold under the same name (for example whether *Solidago gigantea* or *Solidago canadensis*): *Reynoutria* sp., *Rudbeckia* sp., *Solidago* sp. However, in all cases the species within a genus are similar in biology and habitats they occupy.

### Data collection

Comparison of distances that alien invasive plants travelled according to whether sold on-line and by traditional shops

To compare distances on which invasive alien species were transported when sold via internet and in traditional shops, we chose 50 large shops in Poland in different regions of Poland that carried both internet and traditional sale of alien species listed above, and we asked owners to collect data about customers postal codes, sale objects and volume for both internet and traditional commerce. To find suitable shops we searched Google using the phrase: “sklep ogrodniczy”(garden shop) then Yellow Pages portals (www.yellowpages.pl) and Panorama firm (www.panoramafirm.pl, only in polish), the latter is the oldest and best known source for locating all kinds of business including shops. We also selected shops in such a manner that they were dispersed across the entire country. Twenty eight of the owners of these shops agreed to collect the data. We collected data in 2011 for each species on the geographic location of both the shops and customers, number of purchasers, number of plants sold, their life form (whether seedlings or seeds). Shop keepers were aware that data about plant commerce collected by them would be used for further analyses, PhD dissertation, writing scientific papers and publishing. However, they did not know the specific hypotheses that we planned to test. The shop owners were informed about results and ensured that the database will be de-identified, and that they could withdraw the date they had already donated as well as being assured that their personal data (name of the shop and its location etc.) will not be published anywhere. The goal of collected data was to study plant commerce not the human behaviour, and the research possessed no risk to the participants, meaning that the probability and magnitude of harm or discomfort anticipated in the research are not greater in and of themselves than those ordinarily encountered in daily life or during the performance of routine physical or psychological examinations or tests. The data were de-identified. Moreover this research did not involve vulnerable populations and possessed no risk to the participants and, as such, this research did not require Institutional Review Board approval in Poland.

Data provided by the shop owners allowed us to map and visualize the geographical location of the source and destination of each species. Collecting zip codes and location name is a common sales practice as it allows sellers to target subsequent promotions [24]. We also used location name and zip codes to calculate distances between shops and buyers for both internet and traditional sales. We used centroids of the area of a destination location and zip code to calculate the distance transported. We used Quantum 1.7 Wroclaw GIS system.

### Temporal changes in the number of invasive species sold on the internet auction portal

In order to check if internet commerce of invasive species has increased during last 6 years, we used data from the public available archive of Allegro (see http://allegro.pl), the largest on-line auction portal in Poland, to collect data on the number of auctions of the selected invasive alien species. We were able to retrieve data from years 2006–2011. We preferred Allegro as a database instead of the previously chosen sample of shops, because we were interested in (i) estimating the number of all cases of the selected alien invasive species for sale in the entire Poland and (ii) number of purchases made by as many as possible customers in the same period. Moreover, the garden shops had no accurate data on the amount of the two types of sale from past 6 years (or had not recorded it) and some of shops had existed for short period of time. The data were public, thus collecting it did not require Institutional Review Board approval.

### Data processing and analysis

Distances travelled by alien plants through the different purchasing routes were compared using a general linear mixed model; plant species and shop identity were assigned as random factors. A general linear mixed model is an extension to the general linear model in which the linear predictor contains random effects in addition to the usual fixed effects. They are particularly useful in settings where repeated measurements are made on the same statistical units (for example species in our study), or where measurements are made on clusters of related statistical units (shops in this study). Because of their advantages in dealing with data dependency, mixed effects models are often preferred over more traditional approaches, such as repeated measures ANOVA [25]. Random factors in our analyses account for differences between unspecified traits of species and shops that have not been measured during the study.

We also compared features (skewness and kurtosis) of the distance distribution in both sale types for each plant species. Then, we used paired t-student tests to analyse differences between internet and traditional sales in shops. Skewness is a measure of the degree of asymmetry of a distribution around a mean with zeros indicating symmetrical, positive values for right skewed distributions and negative values for left skewed. Kurtosis measures the degree of peakedness of a distribution. Kurtosis higher and lower than 0 indicate leptokurtic (peaked) distribution and platykurtic (flattened) distribution, respectively.

To compare the distances at which seeds and seedlings were transported in the internet and traditional sale we used general linear mixed model. Sale type (internet vs. traditional), life stage (seeds vs. seedling) and interaction between them were introduced as fixed factors. Species identity and shop identity were random effects. In this model only species with both seeds and seedlings in shops’ offer were included.

We used correlation analysis to assess the statistical significance of the temporal trend in the number of internet auctions and number of purchasers in Allegro portal. All statistical analyses were done in SPSS 19.

## Results

### Comparison of distances that alien invasive plants travelled according to whether sold on-line and from traditional shops

For all species the mean distance from a shop to a purchaser was significantly greater when alien invasive plants were sold through the internet than from traditional trade in shops (GLMM *F*_1, 4048_ = 42.58, *P*<0.001, Fig. 1). The effect was consistent among species (Fig. 1). Figure 2 maps, for three species, the distances plants were transported according to whether sold on the internet or in traditional sales; maps for the other species, together with distance distributions, are in Figures S1–S23 in File S1. Also shapes of distributions of distances covered by plants differed between internet and traditional sales. Mean skewness of distance distribution of plants sold via internet was lower than for plants sold in traditional shops (paired *t*-tests: *t*_12_ *= *5.40, *P*<0.001, Fig. 3). The kurtosis coefficient for distance distribution of species sold via internet was lower than in plants sold in a traditional manner (paired *t*-tests: *t*_12_ = 3.71, *P* = 0.003, Fig. 3).

**Figure 1:**
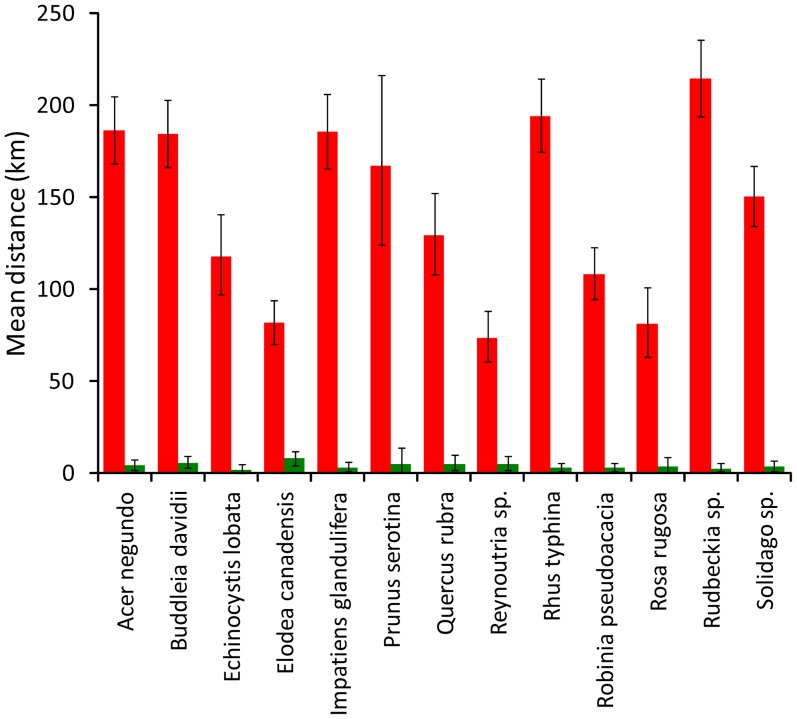
Mean transportation distances for all 13 species traded online (red bars) and in traditional sales (green bars). Whiskers are 95% confidence intervals.

**Figure 2:**
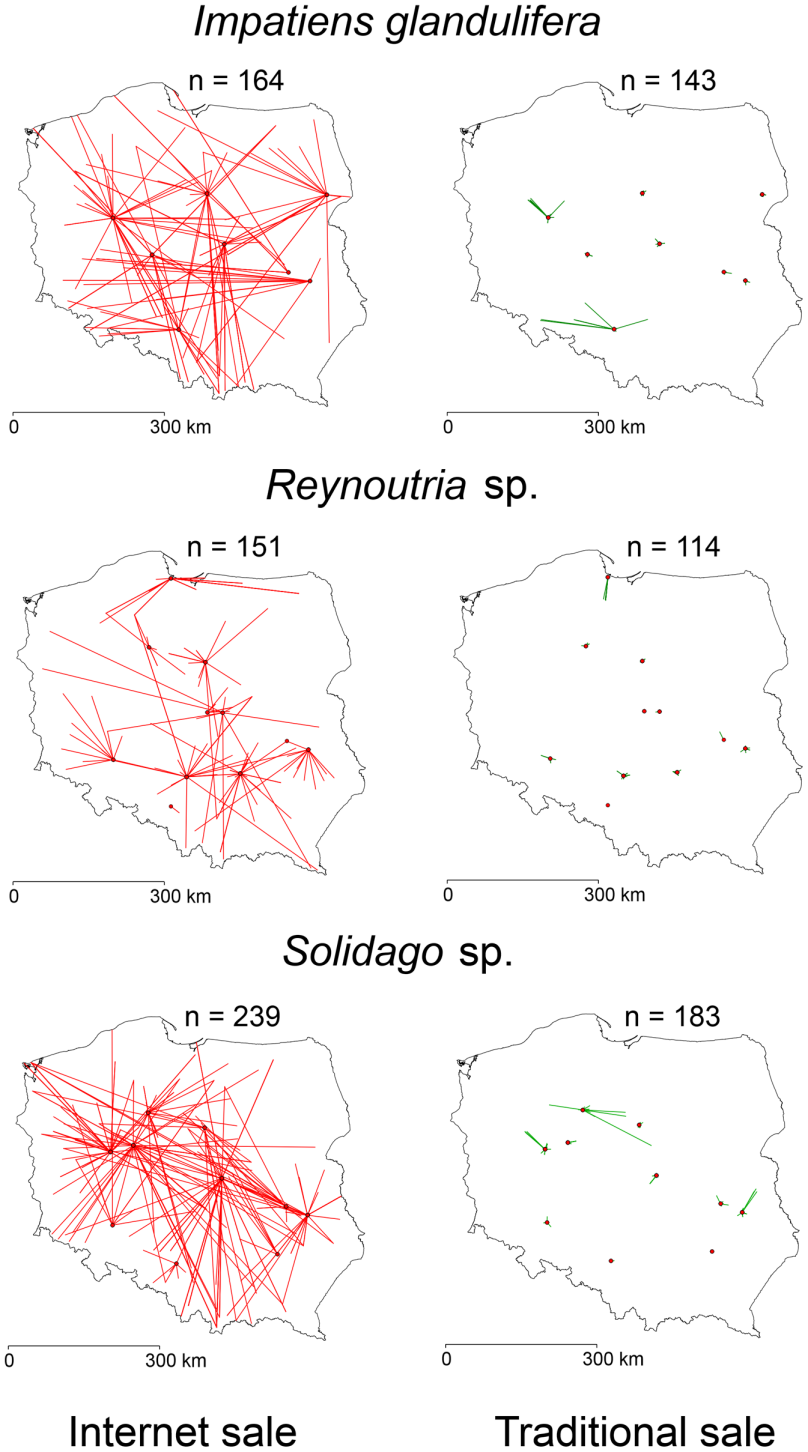
Examples of movement patterns of plants according to the sale type. *Impatiens glandulifera* (a, b), *Reynoutria sp.* (c, d) and *Solidago sp.* (e, f). Red lines indicate distances in the internet trade and green lines in traditional trade. Red dots denote shops locations.

**Figure 3:**
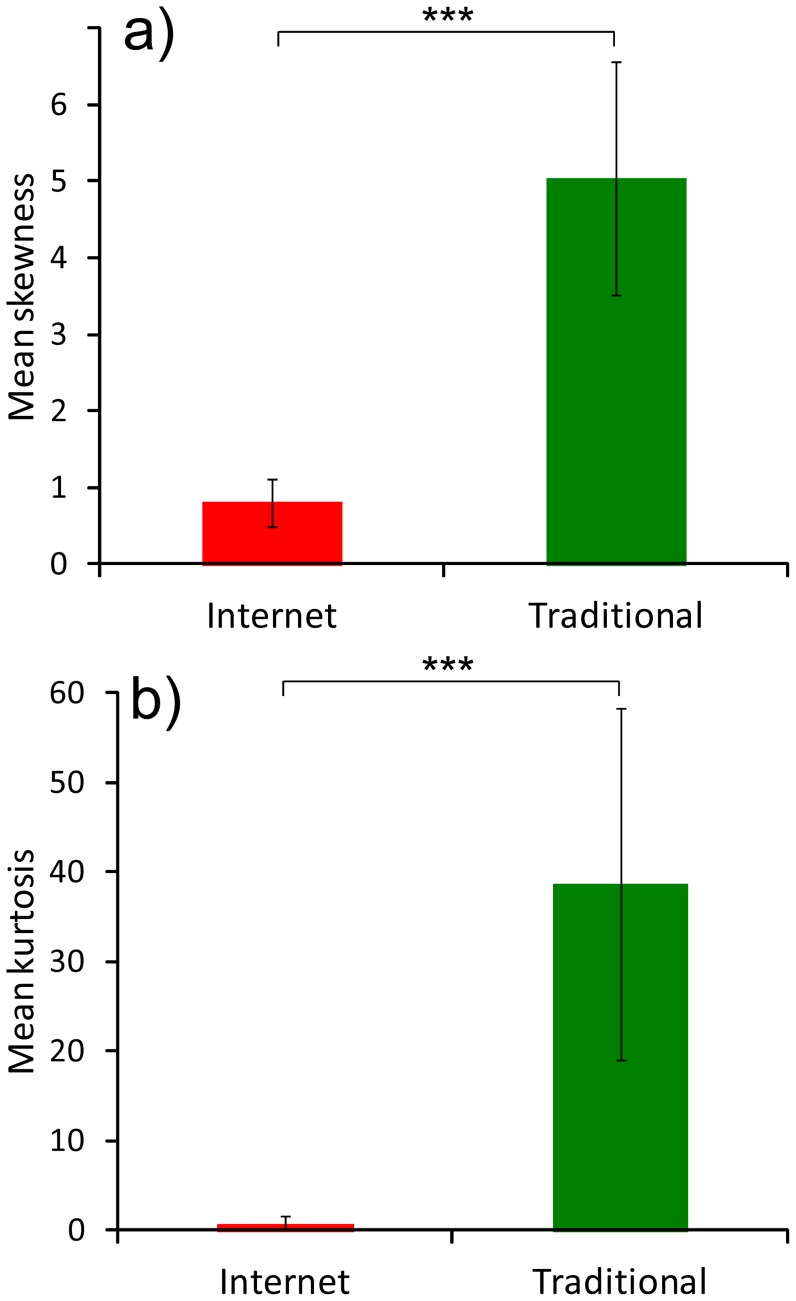
Mean skewness (a) and kurtosis coefficient (b) of distance distribution of invasive plant species traded on-line (red bars) and in a traditional manner (green bars). Explanations: *** - P<0.001. Whiskers are 95% confidence intervals.

**Figure 4:**
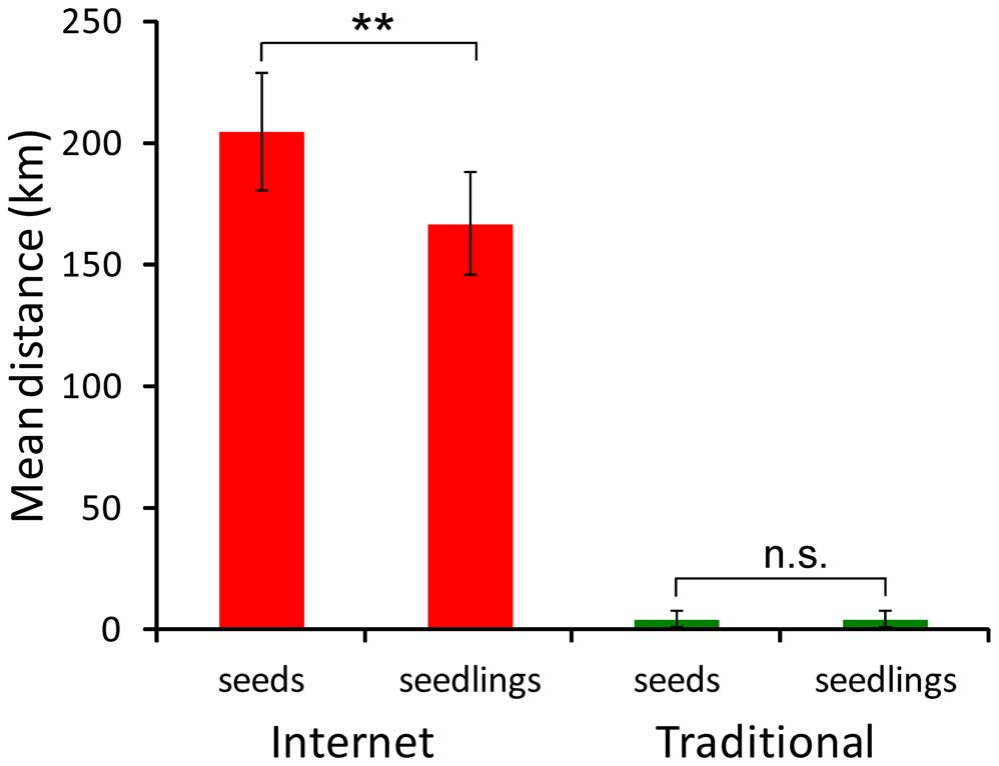
Mean transportation distances of seeds and seedling traded on-line (red bars) and in a traditional manner (green bars). Explanations: ** - P = 0.010; n.s. – statistically non-significant difference. Whiskers are 95% confidence intervals.

The total number of records of purchases from the 28 analyzed shops was 4,050. The number of records involving purchasing seeds was 811 and 3,239 for seedlings. The total number of seeds sold was at least 62,646, and 6,894 for seedlings (in several transactions the number of the plants sold was not specified). For internet commerce the mean transportation distances of seeds were significantly higher (interaction term in GLMM *F*_1, 1990_ = 13.85, *P*<0.001 with Tukey post hoc test *P*<0.010) than for seedlings (Fig. 4) but this was not the case for traditional sales (Fig. 4).

### Temporal changes in the number of invasive species sold on the internet auction portal

The total sale of 13 selected invasive alien species in the largest Polish internet portal increased over time whether measured as total number of purchasers (r = 0.98; *P*<0.001) or auctions (r = 0.99; *P*<0.001) (Fig. 5). Both the number of purchasers and the number of auctions increased for most species studied (Figures S24–S36 in File S1).

**Figure 5:**
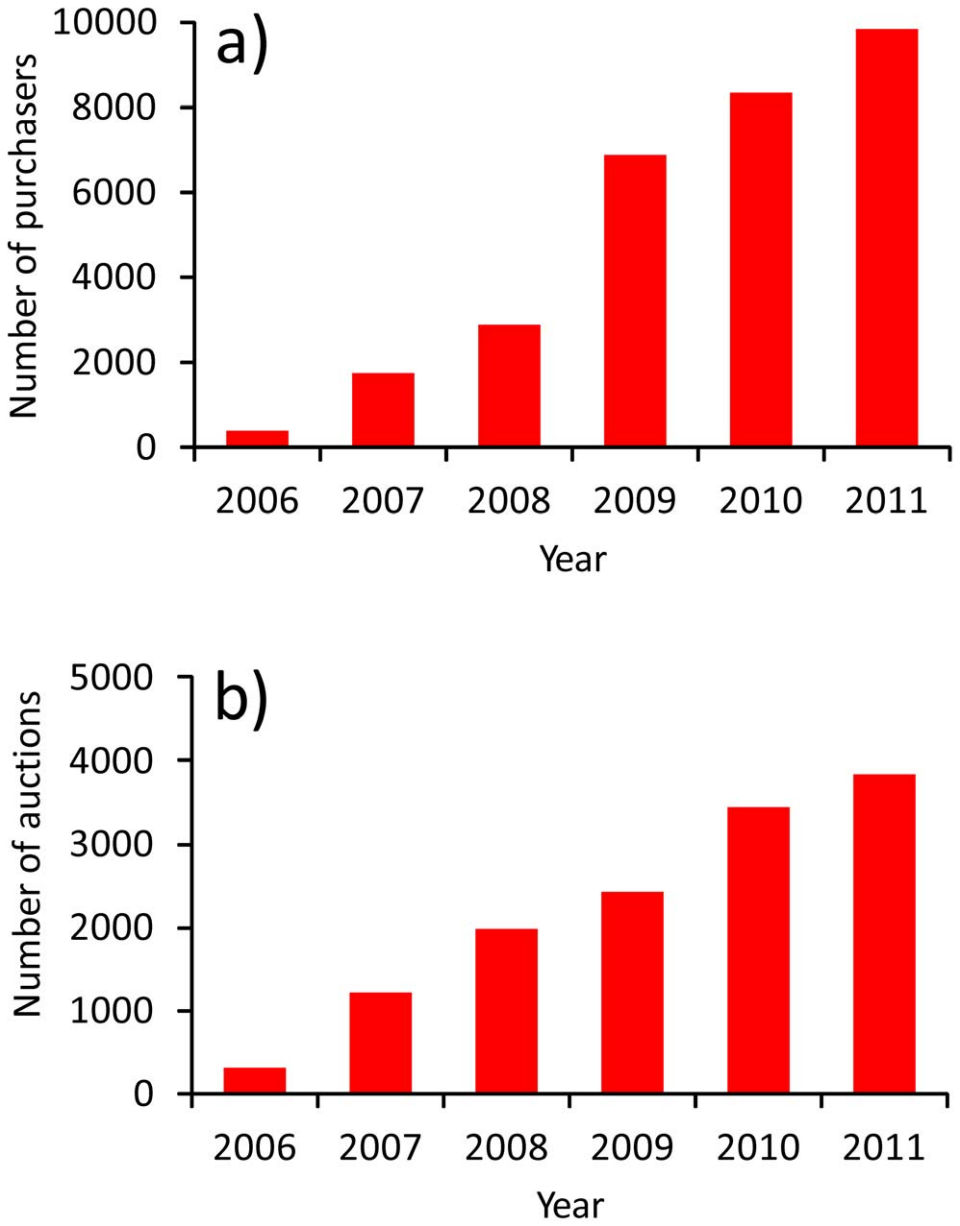
Number of purchasers (a) and number of auctions (b) of 13 invasive alien species in different years in the largest polish internet auctioning portal.

## Discussion

Only 40 years of the internet persistence was necessary for it to become the fastest and the most effective way of communication, but also a very popular means of purchasing goods. Our study shows that socioeconomical changes, especially e-commerce and accessible transport for long distances, modifies dispersal patterns of invasive alien plant species.

Our results clearly show that the distances that invasive species were transported were several times larger if they were ordered by internet than for traditional sales. This result has important ramifications for the understanding invasion processes and indicates that the internet sale generates frequent long-distance dispersal events. Although such long-distance dispersal plays prominent role in invasion ecology and biology of plants [10], [26], natural vectors rarely achieve comparable distances in terrestrial landscapes as the dispersal pattern generated by the internet sale. Therefore such innovative means of species purchasing may play a unique role in alien species invasions. It is believed that the increasing number of the invasions of many groups of organisms is tied to the frequent migration or movements of humans and facilitated long-distance transport [15], [27]–[30]. Also, frequent long distance dispersal is one of the most important factors triggering alien species from the lag phase into population expansion [31]–[33]. The role of distant transport in vehicles and plains has been already recognized, however such cases are rare and incidental and usually represents only tail of the longest dispersal distances. The transport and long-distance dispersal events caused by internet sale are regular and become increasingly frequent. Moreover, the shape of distribution of the distances covered by plants sold via internet were flatten and less skewed than in plant sold in a traditional way. The latter was similar to natural dispersal distance distributions, which are highly right skewed and leptokurtic. The distribution of the distances in the internet sale did not resemble any natural (long) distance distribution of dispersing propagules as it was flatten and only little skewed. Thus it is clear that the internet trade generates novel dispersal modes of invasive species.

Obviously, natural vectors are, and probably will continue to be, important in the spread of alien species. Wind, native dispersers and floods may enhance dispersal of alien species [34]–[37], especially in more traditional landscapes and regions or countries where internet sale is still not well developed. However, if the development of internet web progress these regions will be threaten by invasions of alien species. One of the gaps in understanding consequences of plant long distance dispersal, in general, is that different life stages (seeds, seedlings, adults) may disperse, but documented examples are scarce [10], [38]. This phenomenon was apparent even in the internet sale. In our study seeds were transported on longer distances than seedlings in the internet and this pattern was not shown in traditional sales, probably because purchasers considered seedlings less suitable for long distance postal trade. These differences are likely to have implications for the pattern of use and the probability of establishment. Although seedlings covered slightly shorter distances than seeds the probability of their survival and growth is possibly higher because they are already developed young plants. Usually, only certain proportion of seeds germinate and grow (in a commercial sale it is usually no less than 80%). However, the amount of seeds sold was nine times larger than seedlings indicating that this life form of alien species may be responsible for colonization of new areas and spread. Moreover, seeds may be sometimes lost during transportation adding to occasional spread of alien species outside the gardens (e.g. at road verges) [39].

The frequency with which alien species are transported along a route and delivered to a specific location is strongly associated with the success of dispersion, colonization and subsequent invasion [40]–[42]. Such human related long distance dispersal and increasing propagule pressure enhance genetic variation in populations and help overcome Allee effects and genetic drift that may otherwise reduce invasion success or keeping the invasion process in a very early stage, in a lag phase [43], [44].

Education of potential human vectors is believed to be main factor that can slow down invasions and prevent new ones. However, the example of 13 chosen invasive species, some of the most harmful ones in Europe, shows that they were commonly sold via internet and the number of purchases on the most popular internet portal increased hugely, over one hundred-fold over last few year's (2006–2011). Thus, the internet commerce clearly enhances not only distance of transport but also potentially increase propagule pressure of invasive alien species because customers buying these species usually plant them in gardens so allowing their escape into natural habitats. This growth in sale of aliens seems to reflect general trend in number of internet users that increased globally by 566% between 2000 and 2012 (see http://www.internetworldstats.com) as a result of convenience and time saving provided by the internet communication and internet transactions. The development of the internet sale included numerous alien plant that are not recognized currently as invasive but may become harmful in future. For example, our survey on the internet portals indicated that there are over 300 decorative alien plant species (with different varieties) that have not been recognized as invasive but have being introducing into Poland (Lenda et al. unpublished). This also indicates that ever increasing number of scientific research on invasive species [45] seems not to be effective in preventing invasions and the reason can be poor reflection of professional reports on society education and effectiveness in preventing invasions.

Alien species sold in the internet may become efficient vectors of alien parasites or pathogens also those harmful for native organisms. Much of the spread to the United Kingdom of ash dieback disease *Hymenoscyphus pseudoalbidus* or the sudden oak death ***Phytophthora ramorum*** in USA was attributed to the movement of saplings for forestry [46], [47]. Therefore, the increased number of alien plants being sold, and large distances they are transported via internet sale, amplifies an environmental hazard and creates invasion debt associated with unrecognized pathogens for which aliens species may be just dispersal vectors [48].

Studies in New Zealand show that alien snails, reptile species and alien established frogs were for sale on the internet [22], [48] and that GMO species, forbidden in that country, were also available and were purchased from internet auctions [22], [48]. Finally, internet sales may be a threat for native fauna and flora not only due to commerce of alien invasive species but also by offering many endangered red listed species [21].

### E-commerce contractions and practical recommendation

Countries from European Union, Australia, New Zealand passed legal restrictions (in Poland since 2012) aimed to limit e-commerce of alien and invasive species [22], [48]. However, in all cases sales via the internet still exists, and is even increasing, as sellers may avoid regulations by, for example, changing names of invasive species (like misspelling) or by using very traditional names that are not on police lists (Lenda et al. unpublished).

Solutions to this invasion problem can include increased restrictions, controlling trade, greater enforcement of existing regulations and applying reliable financial fines not only for customers but also for sellers. Further restrictions should limit selling of new alien species or species that are regarded as invasive in other regions of the world. The list of invasive species should be easily accessible for all people and their harmful effects for economy should be estimated in order to caution society about real losses to economy and environment when invasions occur. Sold plants should include information for buyers whether this is an alien or native plant in a given country. More effective reflection of scientific results on invasive species and possible invasion debts on education of society and encouragement for gardeners to grow more benign rather than environmentally damaging plants is desirable.

The growing number of introduced alien species for gardening and agriculture often comes from fashion and follows some recommendations and news in popular media [49]. The society may become much more nature-oriented and aware about environment after watching some nature programs in television or on the internet, or when society follows life-style of some well known, charismatic persons [50]–[53]. Therefore creating a new fashion of planting native plants might be equally important as legal restrictions.

## Conclusions

Our results shed new light on understanding the effects of trade and transportation on spread of alien species by incorporating the increasingly dominating manner of communication between people and societies– the internet. The internet commerce made long distance dispersal very common, which contradicts existing results indicating it is a rare phenomenon. Moreover, internet trade generated shapes of distance distribution that differs from that shown by all other known dispersal vectors. As the number of invasive alien species sold via internet increased over years with the development of e-commerce that may play prominent role for success of alien plants in rapidly changing world.

Thus, a social component of dispersal needs to be included in future dispersal models of alien species. Specifically, further research needs to examine if internet sales affects the spatial pattern of local invasions risks. Buyers' locations may occur in clusters and an examination of socioeconomical factors related to the buyers (such as income, local population size, gross domestic product, education) may provide insights in what drives plant buyers behavior. Ultimately, a better understanding of what drives the buyers of invasive nonnative species may lead to insights in how to manage and limit invasive alien species spread.

## Supporting Information

File S1 Detailed data on distances on which invasive alien were transported when sold on-line and in traditional way in studied garden shops (Figures S1–S23), and the rate of ecommerce for these species in popular Polish auctioning internet portal (Figures S24–S36).
(DOCX)
