# Supplementary material for: Effect of the Internet Commerce on Dispersal Modes of Invasive Alien Species
Source: PLoS One. 2014 Jun 16;9(6):e99786. doi: 10.1371/journal.pone.0099786 (PMC4059692; doi:10.1371/journal.pone.0099786)
Supplement: File S1 — Detailed data on distances on which invasive alien were transported when sold on-line and in traditional way in studied garden shops (Figures S1–S23), and the rate of ecommerce for these species in popular Polish auctioning internet portal (Figures S24–S36). (DOCX) [file pone.0099786.s001.docx]

**Detailed data on distances on which invasive alien were transported when sold on-line and in traditional way in studied garden shops.**

**1. *Acer negundo***

**
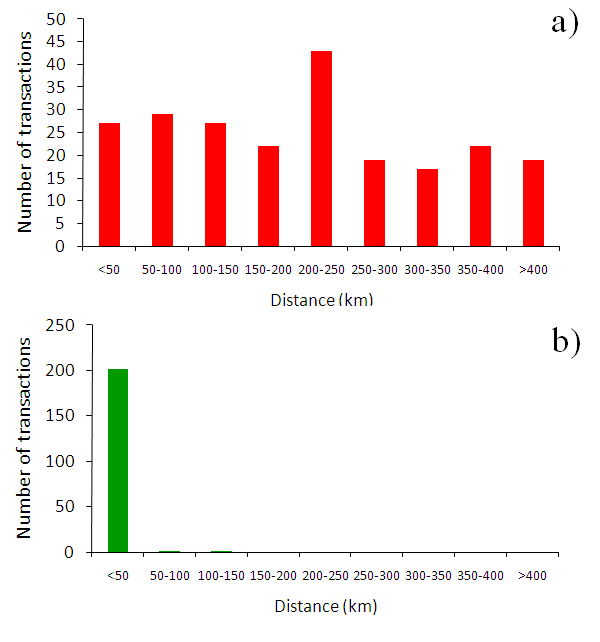
**

**Figure S1.** The distribution of the transportation distances of *Acer negundo* in (a) the internet and (b) traditional sale in studied garden shops.

**
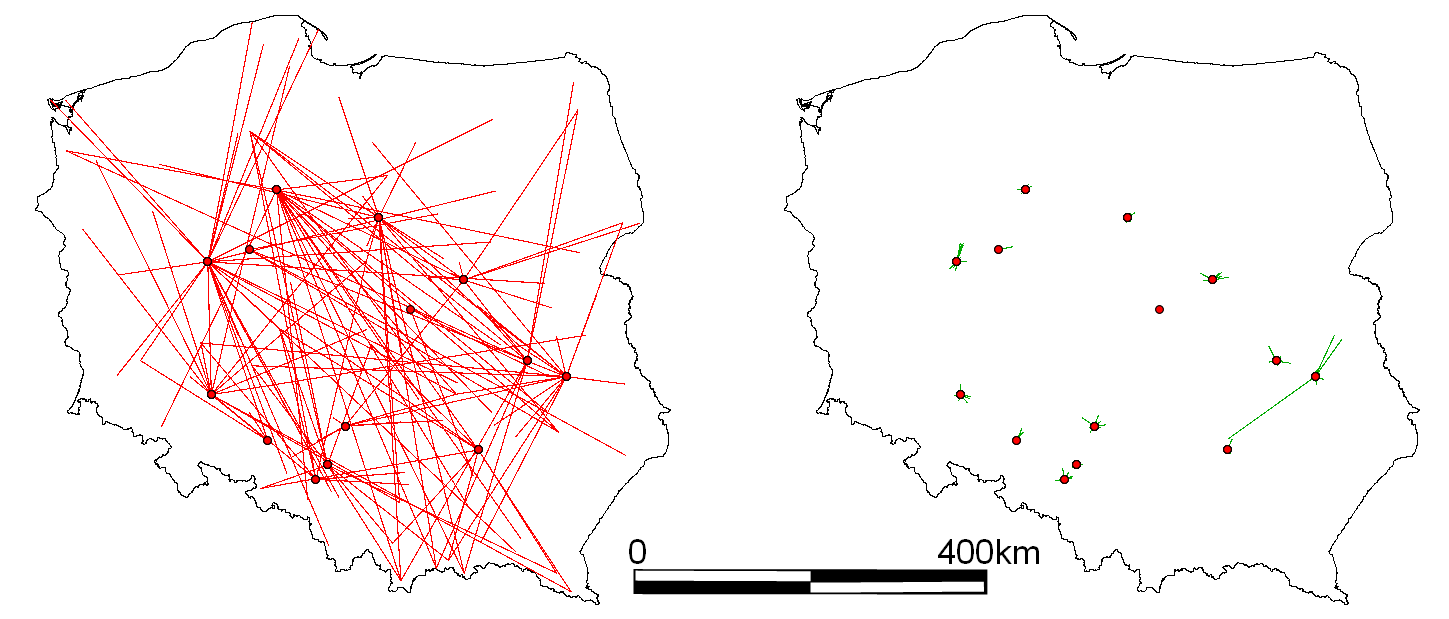
**

**Figure S2.** Maps of distances on which plants were transported depending on the sale type in *Acer negundo.* Red lines indicate distances in the internet trade and green lines in a traditional trade. Red dots denote locations of garden shops.

**2. *Buddleia davidii***

*
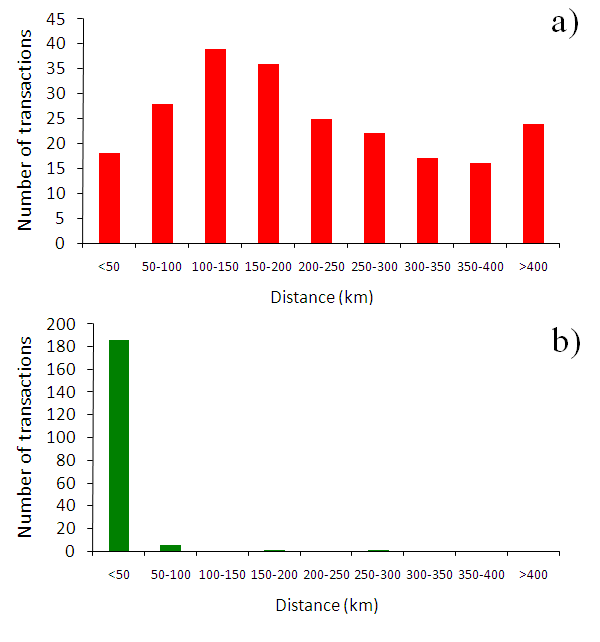
*

**Figure S3.** The distribution of the transportation distances of *Buddleia davidii* in (a) the internet and (b) traditional sale in studied garden shops.

*
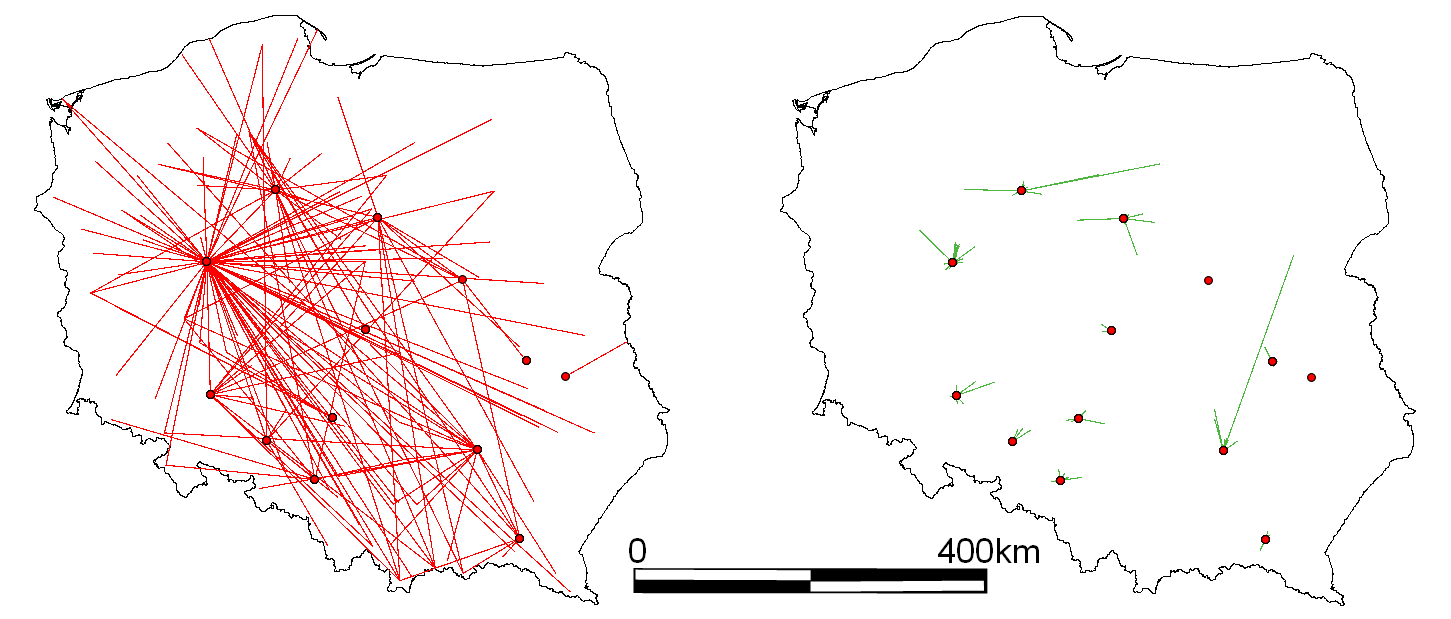
*

**Figure S4.** Maps of distances on which plants were transported depending on the sale type in *Buddleia davidii.* Red lines indicate distances in the internet trade and green lines in a traditional trade. Red dots denote locations of garden shops.

***3. Echinocystis lobata***


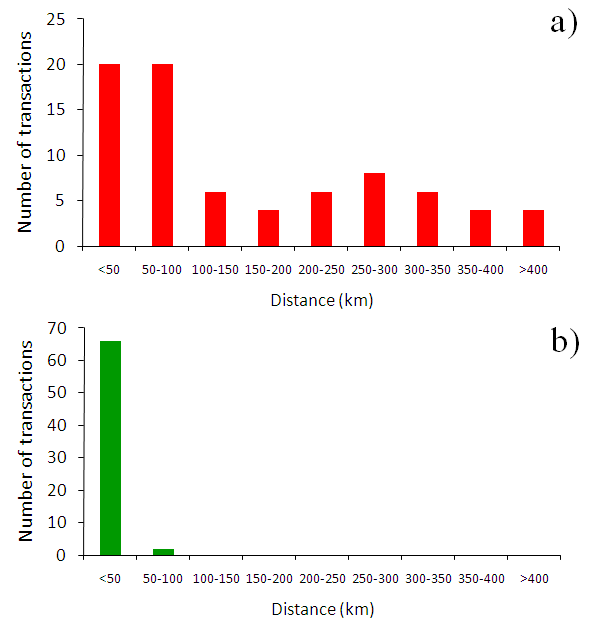


**Figure S5.** The distribution of the transportation distances of *Echinocystis lobata* in (a) the internet and (b) traditional sale in studied garden shops.


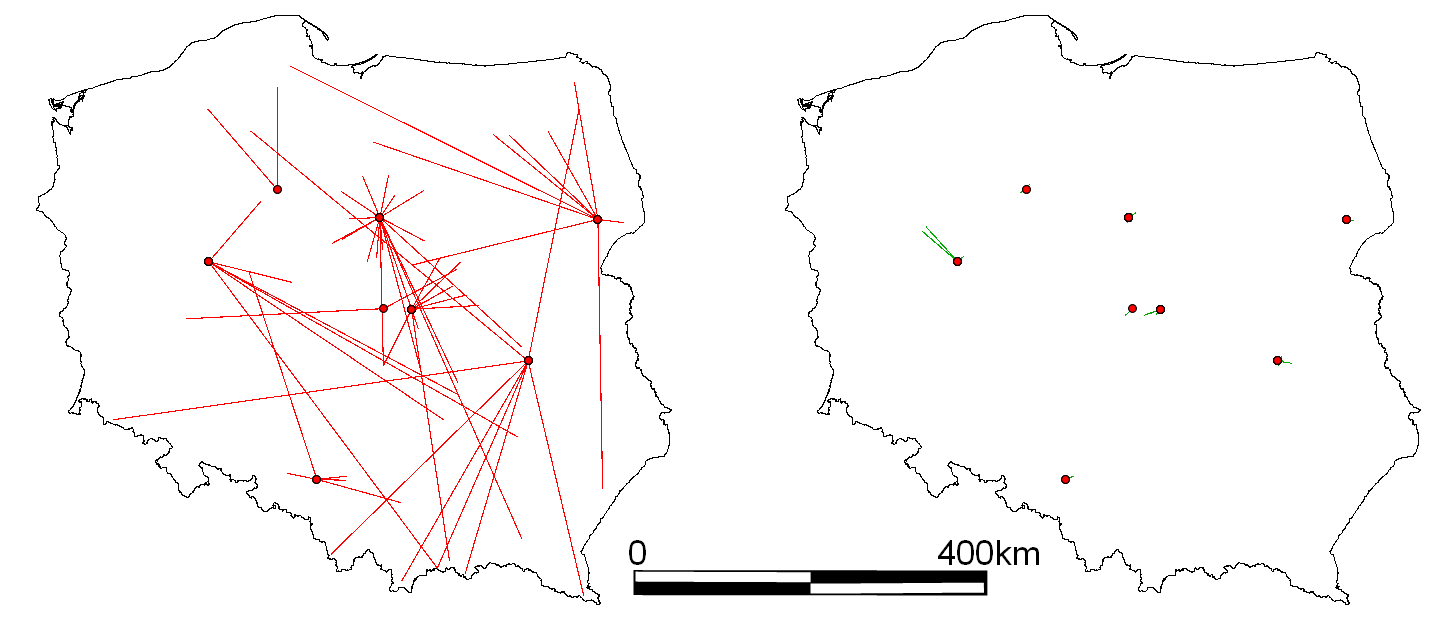


**Figure S6.** Maps of distances on which plants were transported depending on the sale type in *Echinocystis lobata.* Red lines indicate distances in the internet trade and green lines in a traditional trade. Red dots denote locations of garden shops.

**4. *Elodea canadensis***


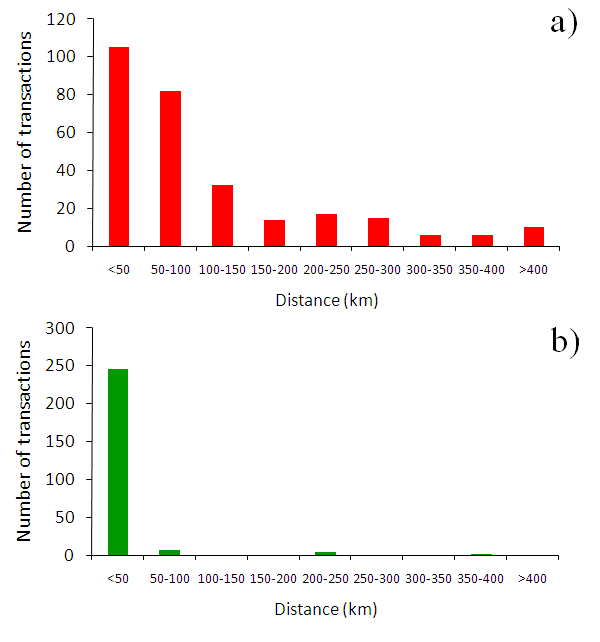


**Figure S7**. The distribution of the transportation distances of *Elodea canadensis* in (a) the internet and (b) traditional sale in studied garden shops.


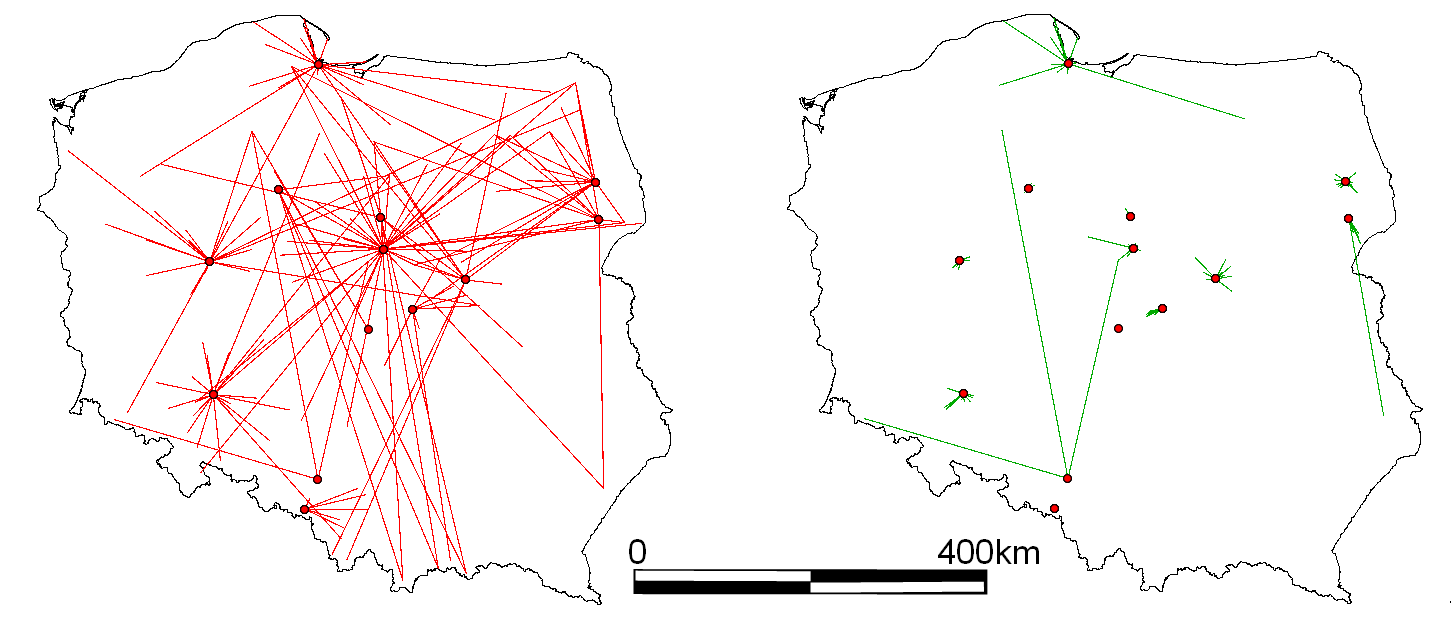


**Figure S8.** Maps of distances on which plants were transported depending on the sale type in *Elodea canadensis.* Red lines indicate distances in the internet trade and green lines in a traditional trade. Red dots denote locations of garden shops.

**5. *Impatiens glandulifera***


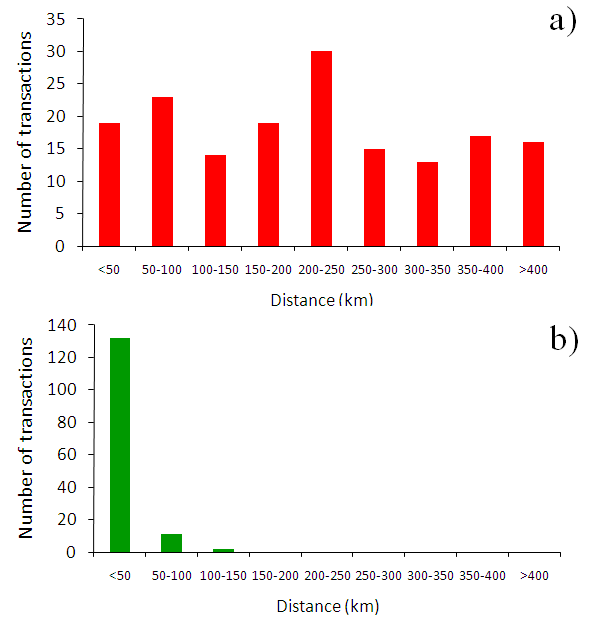


**Figure S9.** The distribution of the transportation distances of *Impatiens glandulifera* in (a) the internet and (b) traditional sale in studied garden shops.

**6. *Prunus serotina***


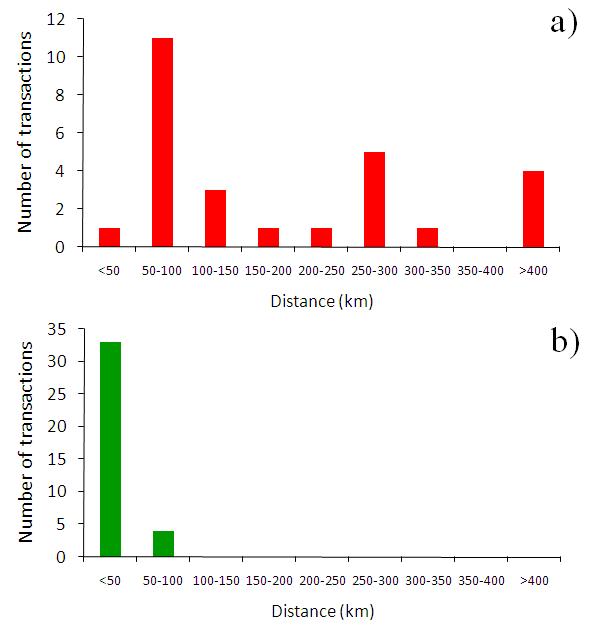


**Figure S10.** The distribution of the transportation distances of *Prunus serotina* in (a) the internet and (b) traditional sale in studied garden shops.


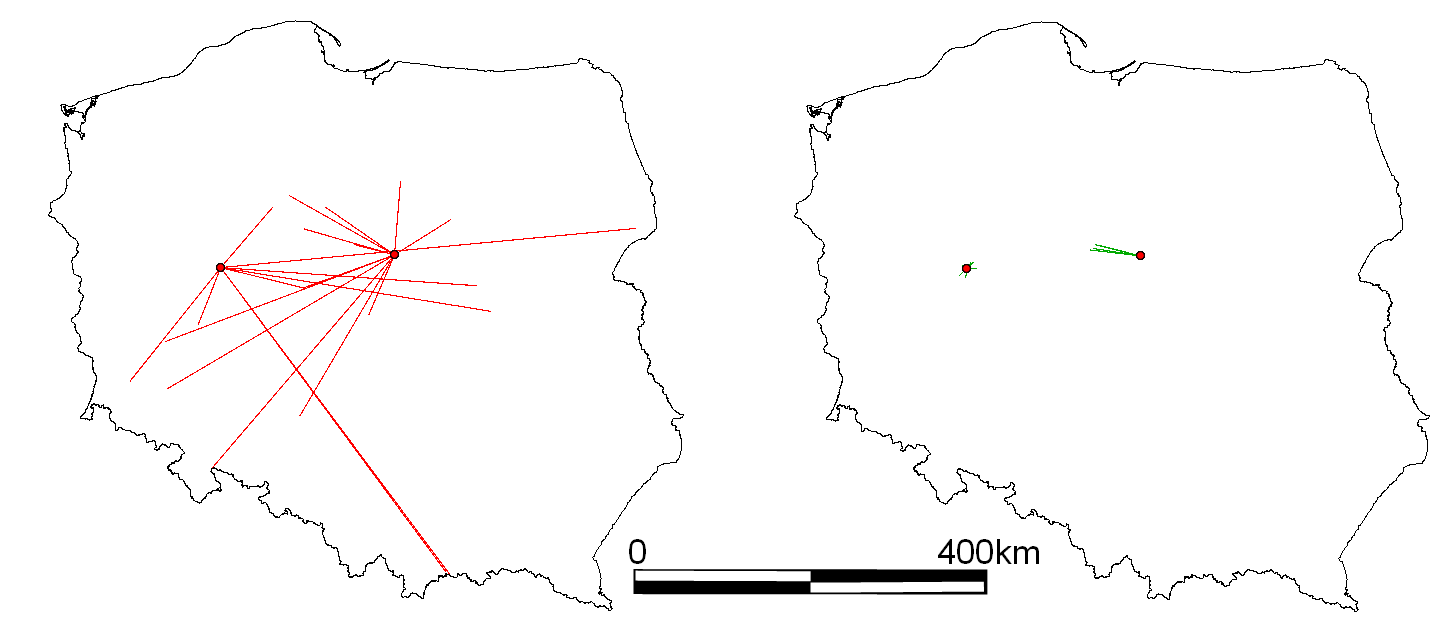


**Figure S11.** Maps of distances on which plants were transported depending on the sale type in *Prunus serotina.* Red lines indicate distances in the internet trade and green lines in a traditional trade. Red dots denote locations of garden shops.

**7. *Quercus rubra***


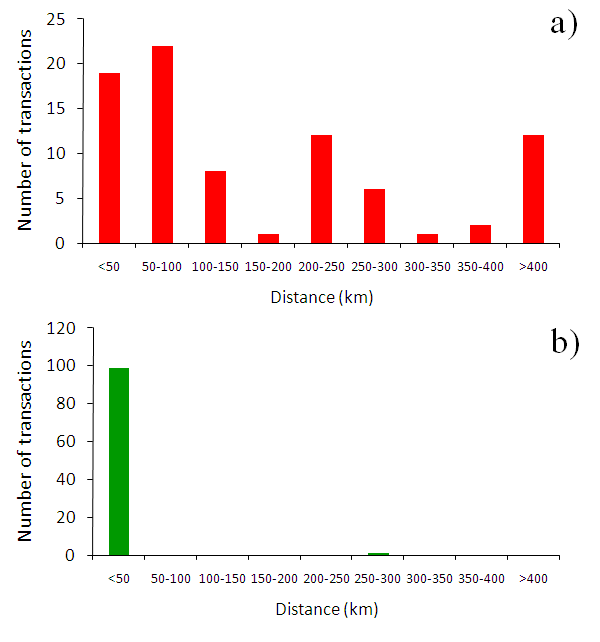


**Figure S12.** The distribution of the transportation distances of *Quercus rubra* in (a) the internet and (b) traditional sale in studied garden shops.


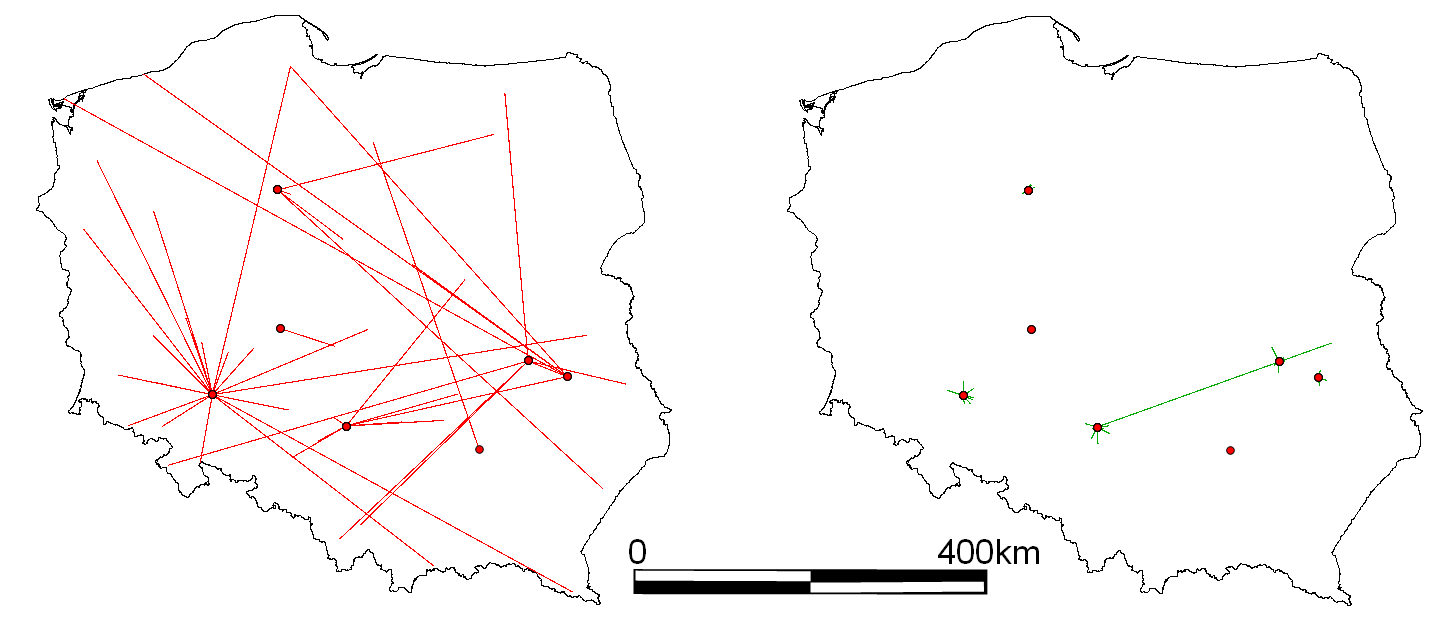


**Figure S13.** Maps of distances on which plants were transported depending on the sale type in *Quercus rubra.* Red lines indicate distances in the internet trade and green lines in a traditional trade. Red dots denote locations of garden shops.

**8. *Reynoutria* sp.**

*
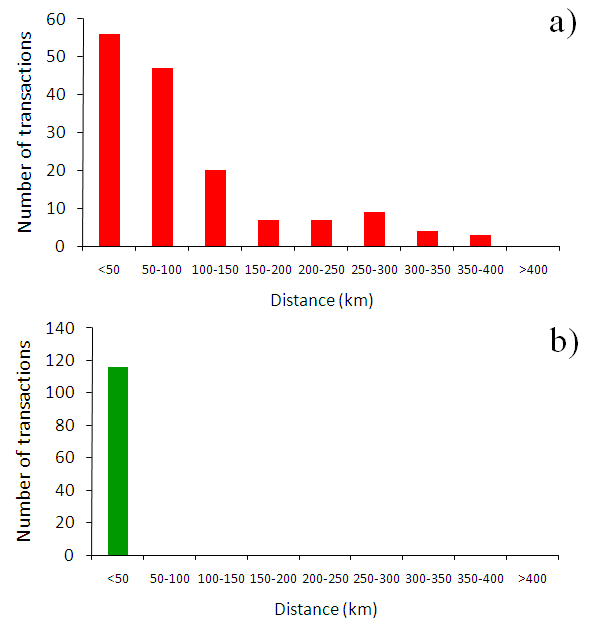
*

**Figure S14.** The distribution of the transportation distances of *Reynoutria* sp. in (a) the internet and (b) traditional sale in studied garden shops.

**9. *Rhus typhina***


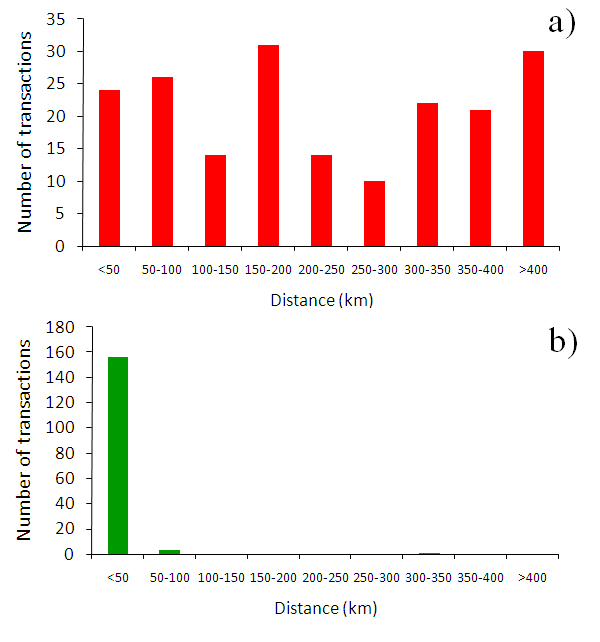


**Figure S15.** The distribution of the transportation distances of *Rhus typhina* in (a) the internet and (b) traditional sale in studied garden shops.


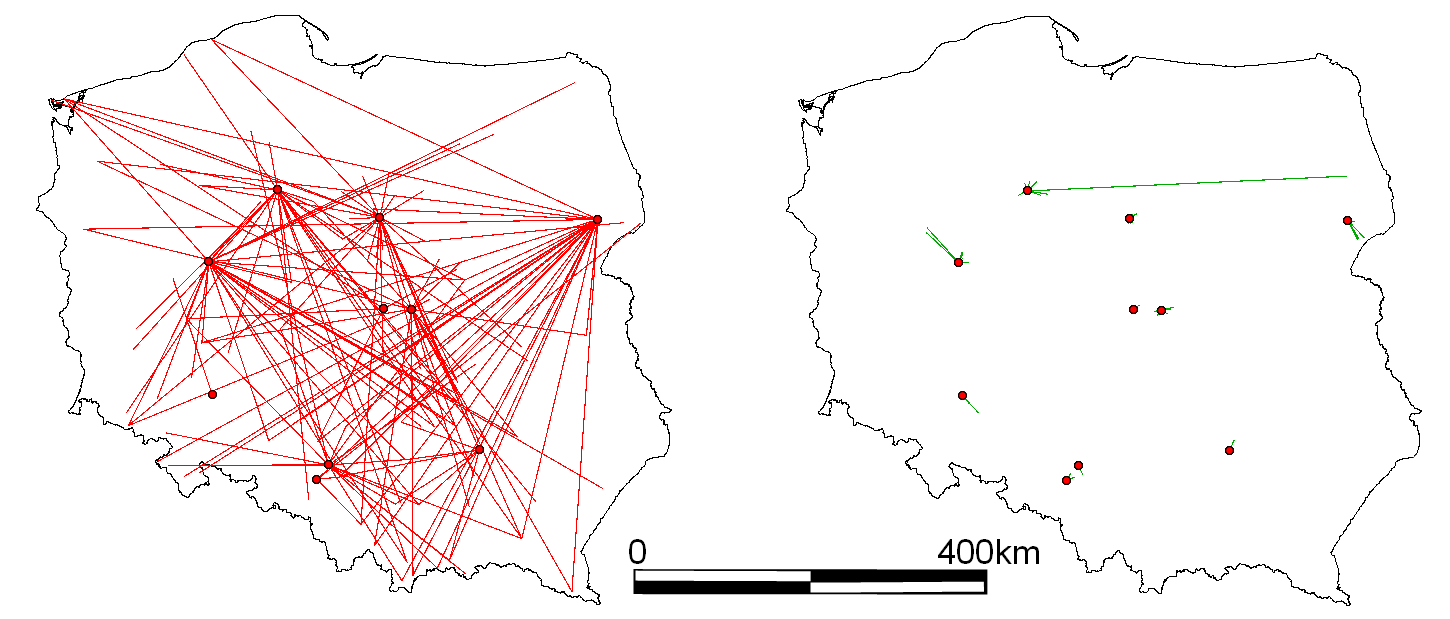


**Figure S16.** Maps of distances on which plants were transported depending on the sale type in *Rhus typhina.* Red lines indicate distances in the internet trade and green lines in a traditional trade. Red dots denote locations of garden shops.

**10. *Robinia pseudoacacia***


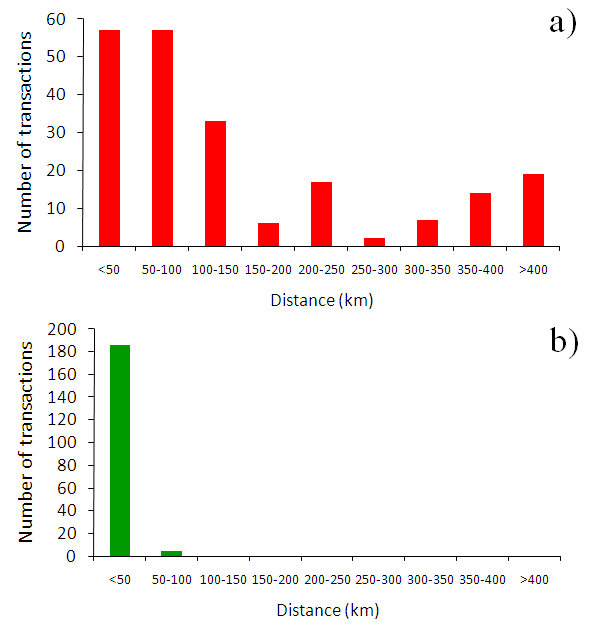


**Figure S17.** The distribution of the transportation distances of *Robinia pseudoacacia* in (a) the internet and (b) traditional sale in studied garden shops.


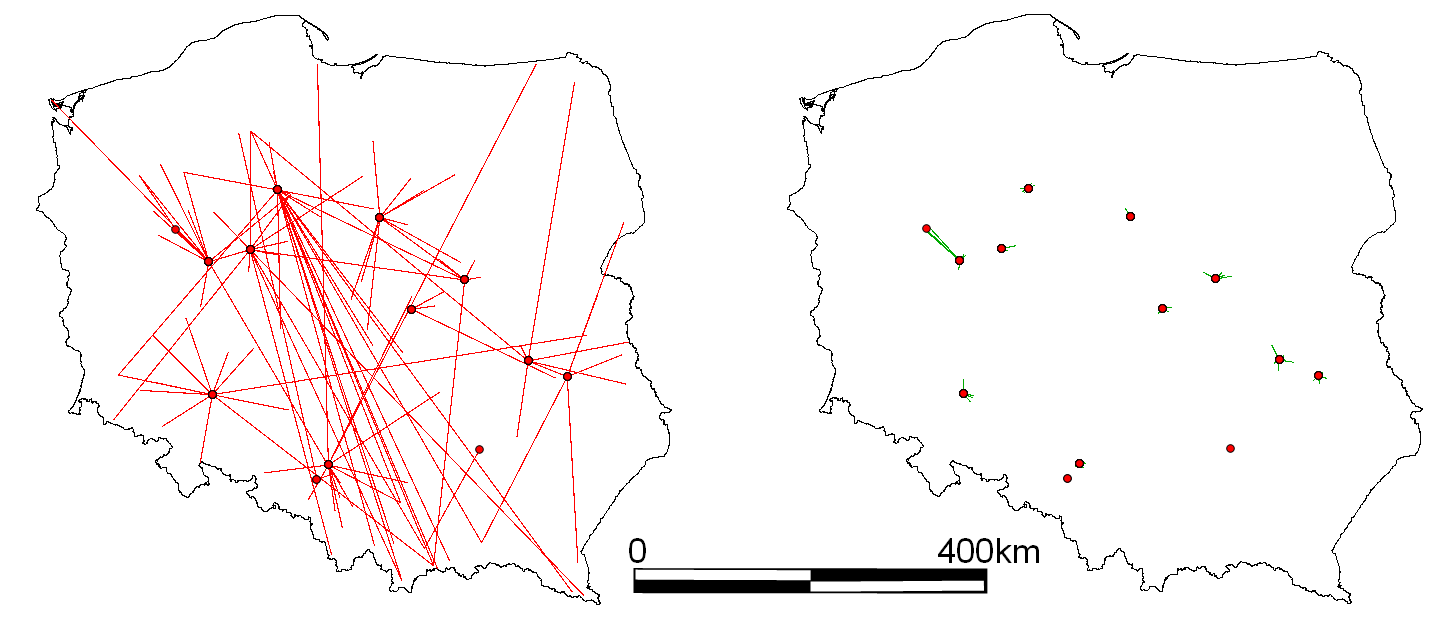


**Figure S18.** Maps of distances on which plants were transported depending on the sale type in *Robinia pseudoacacia.* Red lines indicate distances in the internet trade and green lines in a traditional trade. Red dots denote locations of garden shops.

**11. *Rosa rugosa***


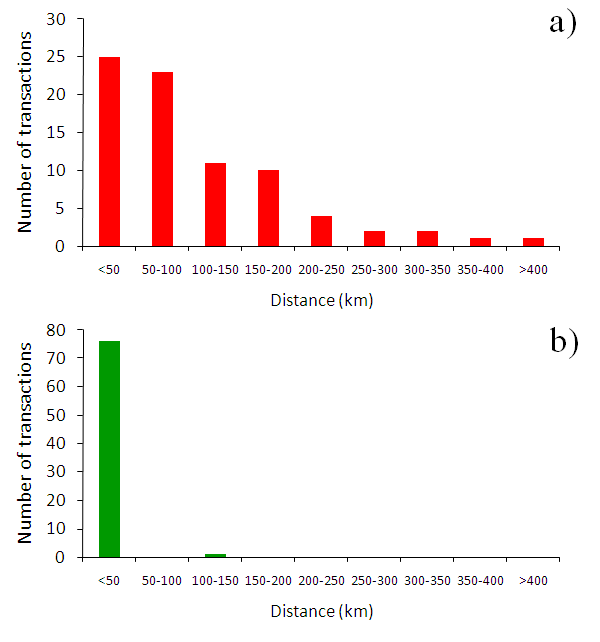


**Figure S19.** The distribution of the transportation distances of *Rosa rugosa* in (a) the internet and (b) traditional sale in studied garden shops.


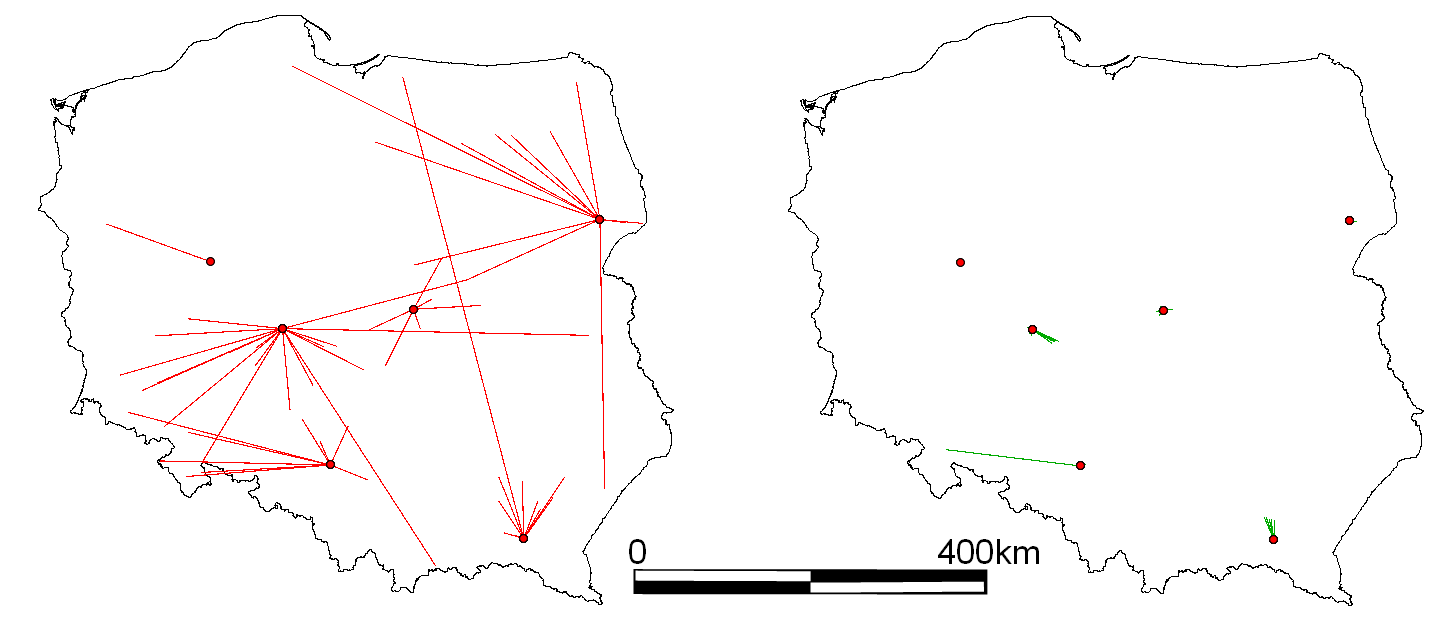


**Figure S20.** Maps of distances on which plants were transported depending on the sale type in *Rosa rugosa.* Red lines indicate distances in the internet trade and green lines in a traditional trade. Red dots denote locations of garden shops.

**12. *Rudbeckia* sp.**


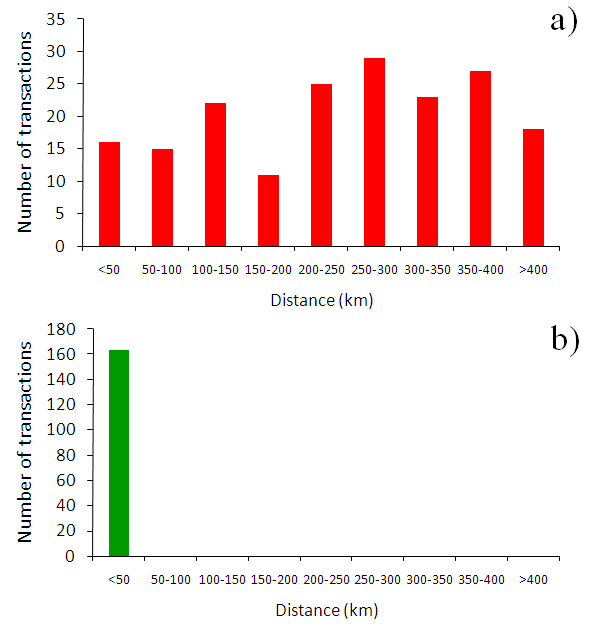


**Figure S21.** The distribution of the transportation distances of *Rudbeckia* sp. in (a) the internet and (b) traditional sale in studied garden shops.


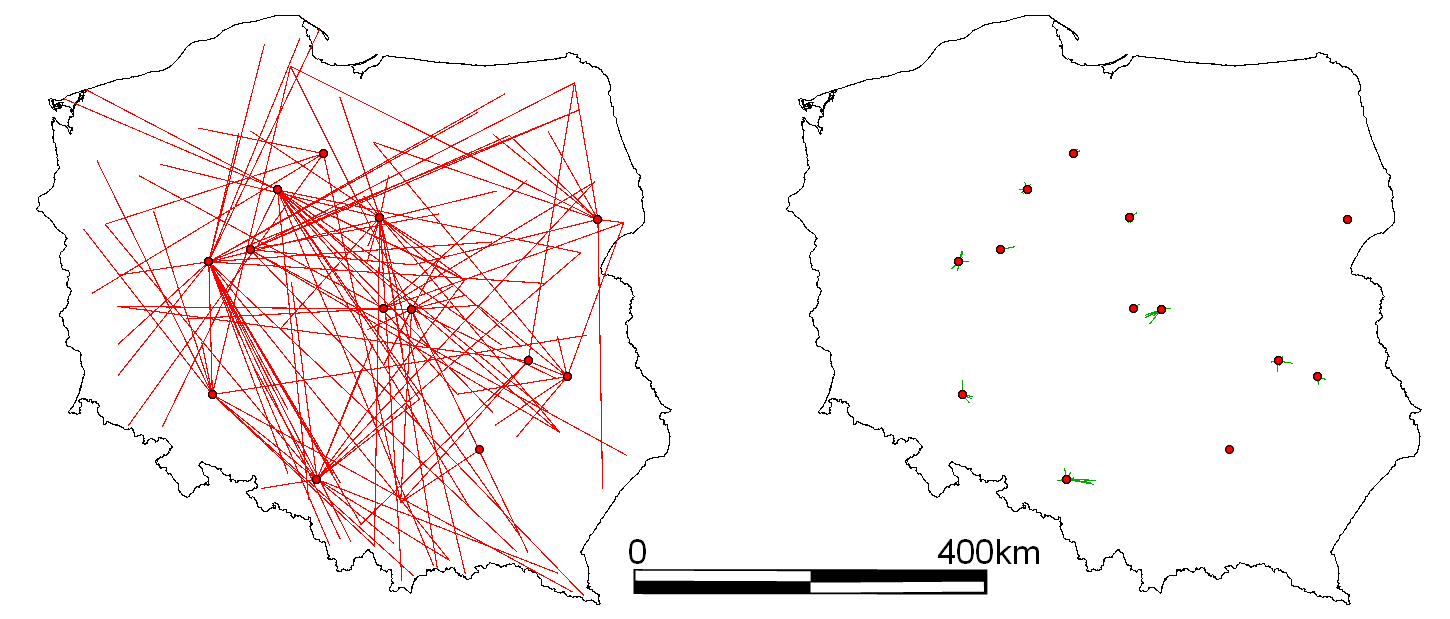


**Figure S22.** Maps of distances on which plants were transported depending on the sale type in *Rudbeckia* sp*.* Red lines indicate distances in the internet trade and green lines in a traditional trade. Red dots denote locations of garden shops.

**13. *Solidago* sp.**

*
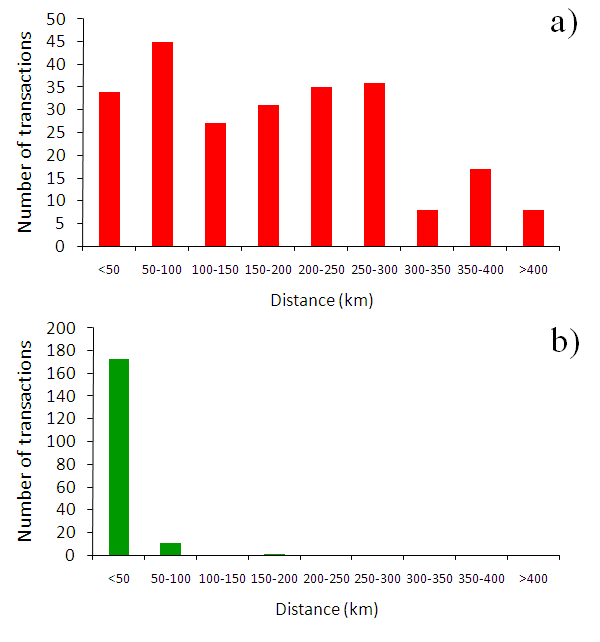
*

**Figure S23.** The distribution of the transportation distances of *Solidago* sp. in (a) the internet and (b) traditional sale in studied garden shops.

**Detailed data on rate of ecommerce for 13 the most harmful invasive species in Europe sold during recent 6 years on the most popular polish auctioning internet portal Allegro**

1. ***Acer negundo***

**
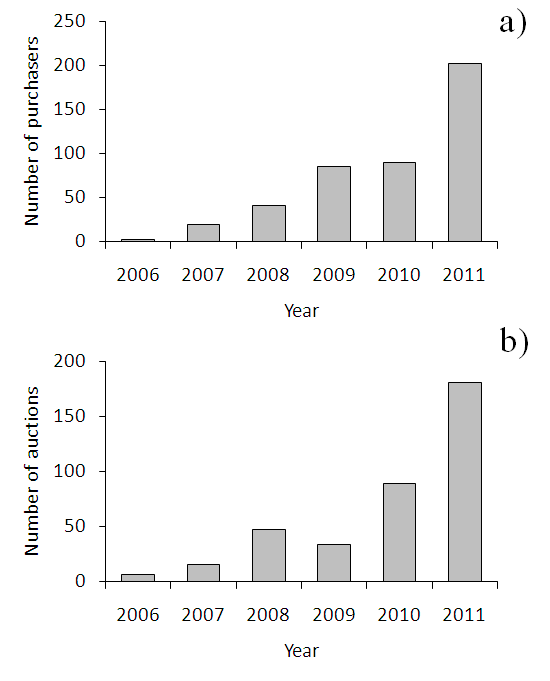
**

**Figure S24.** Number of purchasers (a) and number of auctions (b) of *Acer negundo* in different years in the largest polish internet auctioning portal.

1. ***Buddleia davidii***

*
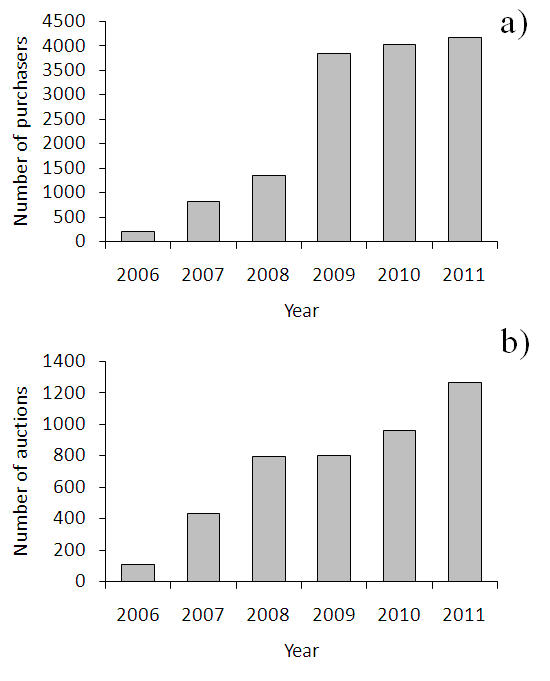
*

**Figure S25.** Number of purchasers (a) and number of auctions (b) of *Buddleia davidii*  in different years in the largest polish internet auctioning portal.

1. ***Echinocystis lobata***

*
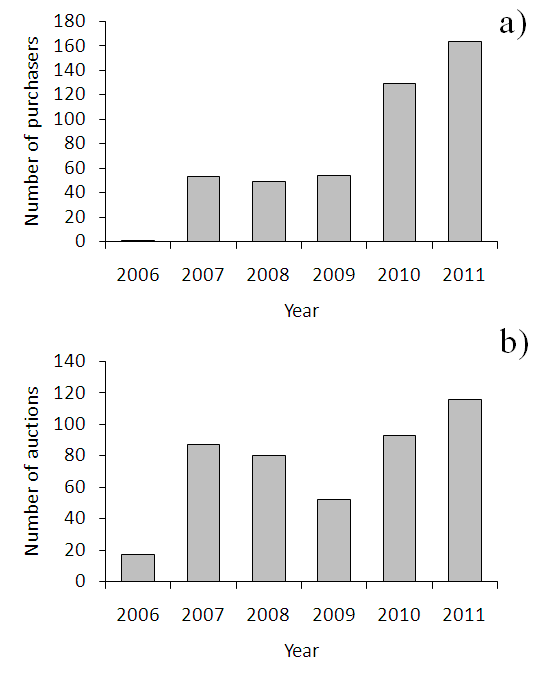
*

**Figure S26.** Number of purchasers (a) and number of auctions (b) of *Echinocystis lobata*  in different years in the largest polish internet auctioning portal.

1. ***Elodea canadensis***


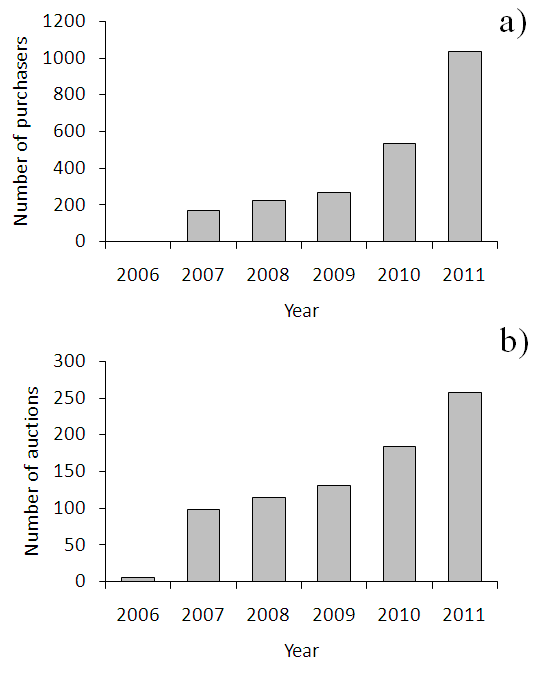


**Figure S27.** Number of purchasers (a) and number of auctions (b) of *Elodea canadensis* in different years in the largest polish internet auctioning portal.

1. ***Impatiens glandulifera***


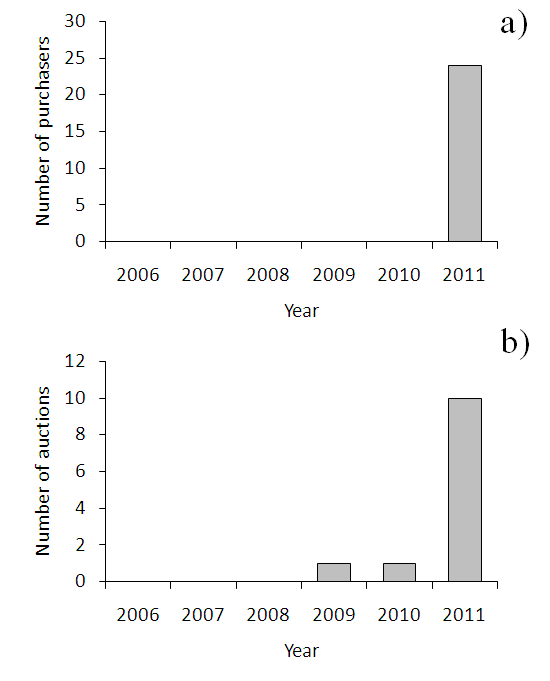


**Figure S28.** Number of purchasers (a) and number of auctions (b) of *Impatiens glandulifera* in different years in the largest polish internet auctioning portal.

1. ***Prunus serotina***


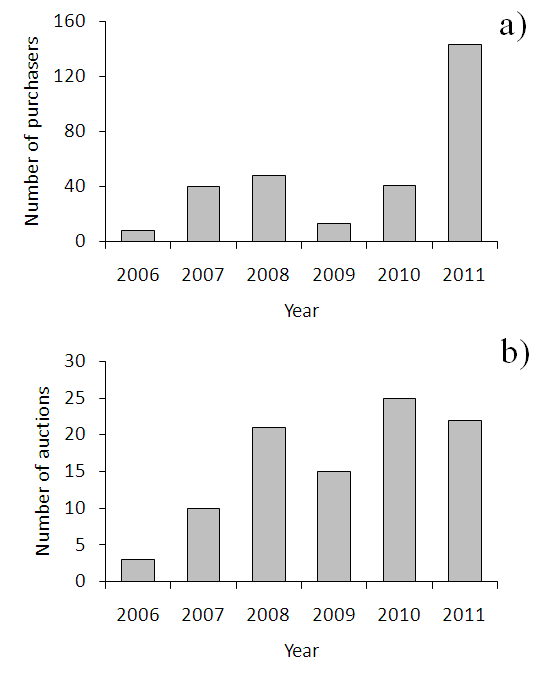


**Figure S29.** Number of purchasers (a) and number of auctions (b) of *Prunus serotina* in different years in the largest polish internet auctioning portal.

1. ***Quercus rubra***


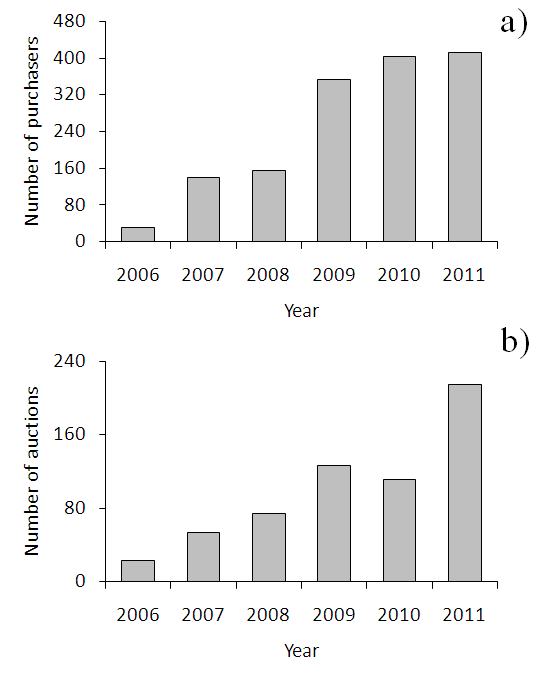


**Figure S30.** Number of purchasers (a) and number of auctions (b) of *Quercus rubra* in different years in the largest polish internet auctioning portal.

1. ***Reynoutria sp.***

*
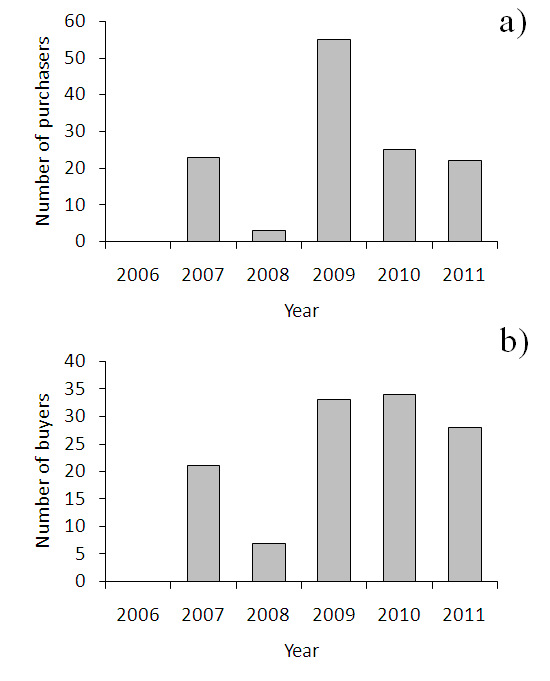
*

**Figure S31.** Number of purchasers (a) and number of auctions (b) of *Reynoutria* sp. in different years in the largest polish internet auctioning portal.

1. ***Rhus typhina***


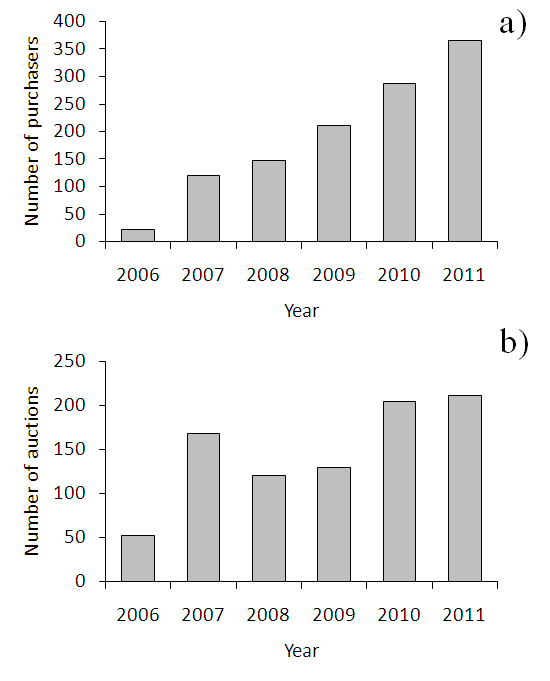


**Figure S32.** Number of purchasers (a) and number of auctions (b) of *Rhus typhina* in different years in the largest polish internet auctioning portal.

1. ***Robinia pseudoacacia***


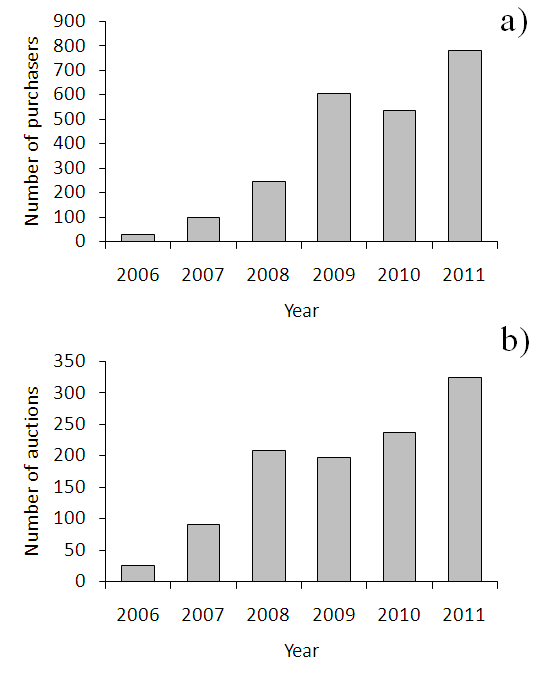


**Figure S33.** Number of purchasers (a) and number of auctions (b) of *Robinia pseudoacacia* in different years in the largest polish internet auctioning portal.

1. ***Rosa rugosa***


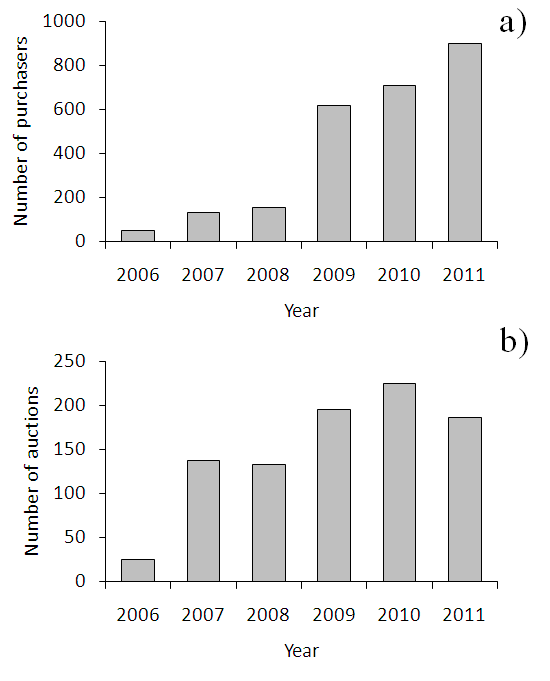


**Figure S34.** Number of purchasers (a) and number of auctions (b) of *Rosa rugosa* in different years in the largest polish internet auctioning portal.

1. ***Rudbeckia* sp*.***


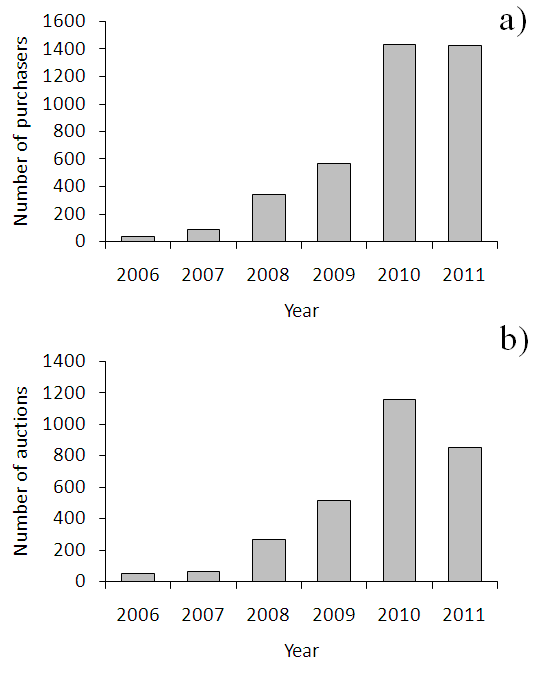


**Figure S35.** Number of purchasers (a) and number of auctions (b) of *Rudbeckia* sp. in different years in the largest polish internet auctioning portal.

1. ***Solidago* sp*.***

*
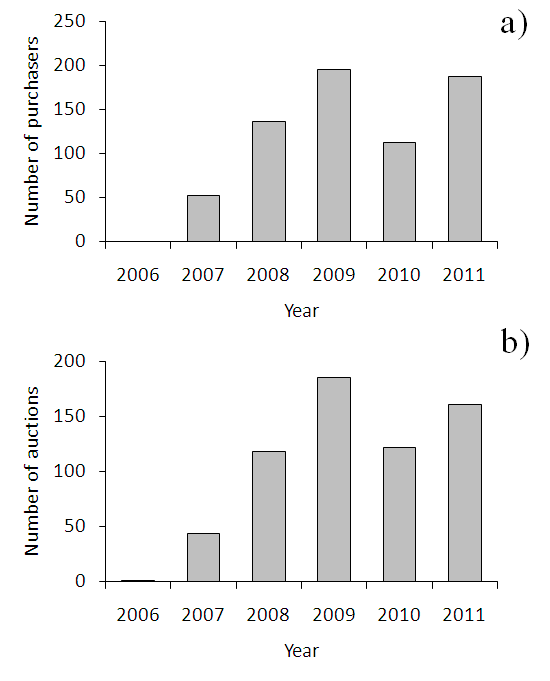
*

**Figure S36.** Number of purchasers (a) and number of auctions (b) of *Solidago* sp. in different years in the largest polish internet auctioning portal.
